# A controlled study to determine the efficacy of *Loxostylis alata* (Anacardiaceae) in the treatment of aspergillus in a chicken (*Gallus domesticus*) model in comparison to ketoconazole

**DOI:** 10.1186/1746-6148-8-210

**Published:** 2012-10-31

**Authors:** Mohammed M Suleiman, Neil Duncan, Jacobus N Eloff, Vinny Naidoo

**Affiliations:** 1 Phytomedicine Programme, Department of Paraclinical Sciences, Faculty of Veterinary Science, University of Pretoria, Pretoria, South Africa; 2 Section of Pathology, Department of Paraclinical Sciences, Faculty of Veterinary Science, University of Pretoria, Pretoria, South Africa; 3 Section of Pharmacology and Toxicology, Department of Paraclinical Sciences, Faculty of Veterinary Science, University of Pretoria, Pretoria, South Africa; 4 Department of Physiology and Pharmacology, Faculty of Veterinary Medicine, Ahmadu Bello University, Zaria, Nigeria

**Keywords:** *Loxostylis alata*, Aspergillosis, Broiler chicks

## Abstract

**Background:**

The poultry industry due to intensive methods of farming is burdened with losses from numerous infectious agents, of which one is the fungus *Aspergillus fumigatus*. In a preliminary study, the extracts of *Loxostylis alata* A. Spreng, ex Rchb. showed good activity *in vitro* against *A. fumigatus* with a minimum inhibitory concentration of 0.07 mg/ml. For this study crude, a crude acetone extract of *L. alata* leaves was evaluated for its acute toxicity in a healthy chicken model and for efficacy in an infectious model of aspergillosis (*A. fumigatus*).

**Results:**

At a dose of 300 mg/kg, the extract induced some toxicity characterised by decreased feed intake and weight loss. Consequently, 100 and 200 mg/kg were used to ascertain efficacy in the infectious model. The plant extract significantly reduced clinical disease in comparison to the control in a dose dependant manner. The extract was as effective as the positive control ketoconazole dosed at 60 mg/kg.

**Conclusions:**

The results indicate that a crude extract of *L. alata* leaves has potential as an antifungal agent to protect poultry against avian aspergillosis.

## Background

The poultry industry which is an important component in world agricultural economy faces heavy economic losses due to many health hazards caused by the fungus *Aspergillus fumigatus*. Losses caused low productivity, mortality and carcass condemnations at slaughter [1-3]. About US$11 million is the reported as average yearly lost due to aspergillosis alone in the USA [3]. The disease affects mainly the respiratory tract of birds and has a worldwide distribution, having been reported in almost every farmed bird as well as in wild species [3].

As a disease, aspergillosis affects birds whether in captive or free-ranging environments, young and mature, and whether immunocompetent or immunosuppressed. However, young birds appear to be much more susceptible than adults. The lower respiratory tract is where *Aspergillus* spp. tends to initially colonize [4] but blood infection with subsequent dissemination to other organs frequently occurs, leading to macroscopic lesions in a wide range of organs or tissues. In spontaneous cases, lesions range from miliary to larger granulomatous foci [5]. These white lesions are protrusive to the surface of the internal organ. Thickening of the walls of the air sacs frequently occurs [6]. Lesions in avian species are commonly confined to the lungs and air sacs, although infections also occurs in oral mucosa, trachea, brain, eye, skin, bone, liver, kidney and nasal passages have also been described [7,8]. Typical lesions are characterized by granulomatous inflammation with necrosis, haemorrhage, and intralesional fungal elements that are locally invasive. The pathogenesis of aspergillosis appears to be complicated. In recent years, aspergillosis has also emerged as a significant disease in humans that are immunocompromised by HIV-AIDS, neoplasia, or chemotherapy [9].

Although there are commercial drugs available for the treatment of systemic and superficial mycoses [10-12], none of them are ideal in terms of availability, efficacy, safety and antifungal spectrum for use by the poultry industry[13,14]. There is therefore a need to explore new remedies to treat the disease especially phytomedicines which may be a more natural means of treating infections. One plant would potential benefit could be *Loxostylis alata* A. Spreng, ex Rchb. (common name tarwood). As a plant Loxostylis is an evergreen, ornamental tree that grows to a height of 5 metre naturally in rocky and forest areas along river banks. The leaves are alternate and compound with 2 to 5 pairs of leaflets, including a terminal leaflet. The fruits of L. alata are small, fleshy and measure about 4 mm in diameter, usually they are found embedded in the brightly coloured sepals. The seed skin contains a black sticky substance like tar; it is difficult to wash when touched [15].

In a preliminary random screening study, this plant demonstrated good in vitro activity against Aspergillus fumigatus and other pathogenic animal fungi with minimum inhibitory concentration (MIC) as low as 0.07 mg/ml [16]. Moreover, bioautography of L. alata extracts with A. fumigatus revealed good antifungal activity [17]. This study aims at evaluating the in vivo effect of L. alata leave extract against experimental aspergillosis in poultry.

## Methods

### Plant collection, extraction and processing

Leaves were collected at the Manie van der Schijff Botanical Garden of the University of Pretoria, South Africa. Samples of the plant were identified and authenticated by Lorraine Middleton and Magda Nel of the University of Pretoria. Voucher specimen of the plant with number; PRU96508 was deposited at the Schweicker Herbarium, University of Pretoria, South Africa. After collection and transportation to the laboratory, leaves were separated from stems and dried at room temperature with good ventilation. The dried leaves were milled to a fine powder in a Macsalab mill (Model 200 LAB, Eriez®, Bramley) and stored at room temperature in closed containers in the dark until used. Five hundred grams of finely ground plant material was extracted with 5 litres of acetone. The extraction process was repeated three times to exhaustively extract the same plant material, and the extracts were combined. The solvent was removed *in vacuo* to yield 75 grams of dark greenish solid. The extract was dissolved in 0.2% aqueous dimethylsulphoxide (DMSO) at a concentration of 200 mg/ml.

### Animals and management

Three day old broiler chicks (Ross 308) were used to evaluate the safety of the extract in a toxicity trial (n=3) and its efficacy (n=8) on birds experimentally infected with *Aspergillus fumigatus*. The birds were purchased from a healthy breeding flock at Eagles Pride Hatchery, Pretoria. Throughout the experiment, broiler chicks were kept at the Poultry Reference Centre, Faculty of Veterinary Science, University of Pretoria in an enclosed temperature-controlled house with adequate non-HEPA ventilation, an artificial light at the recommended light intervals source. Clean wood shaving was used as bedding [18]. Feed intake and weight gain for each group were determined every other day. All experimental protocols described in this study were approved by the Animal Use and Care Committee of the University of Pretoria, South Africa (V036/08) in accordance with the international guidelines for use of animals in experimentation.

### Safety evaluation of the extract

A modified version of the guidelines for Organisation for Economic Cooperation and Development (OECD) for determining the toxic nature of the chemical (Annex 2b; starting dose of 50 mg/kg) was used to determine the dose of the extract that will produce toxic signs and possibly death in treated birds. Toxic signs exhibited such as ruffled feathers, diarrhoea, depression, off-feed, rather than death was used as an end point in determining the safety of the extract. Chicks were randomly assigned into 4 groups of 3 birds each. The first group received the extract at the dose of 300 mg/kg recommended by OECD, while the second group was dosed with 0.2% aqueous dimethylsulphoxide at the dose of 0.2 ml/100 g body weight in water and served as control. All birds were monitored for 12 days. Due to the toxic effect of the starting dose (300 mg/kg), lower doses of 50 and 200 mg/kg of the extract were administered to groups 3 and 4, respectively. These chicks were also examined for signs of toxicity. The dose that did not produce any toxic sign was used as the maximum tolerated dose (MTD) for the chemotherapeutic trial. All treatments were given intraperitoneally with a 23½ G needle attached to a 1ml syringe (TERUMO Medical Corporation, Elkton, MD 21921, USA).

### Experimental inoculum

The *Aspergillus fumigatus* used was isolated from an infected chick airsac on a broiler farm in Gauteng, South Africa by Dr J. Picard and maintained on Sabouraud dextrose agar 6.5% supplemented with 50 mg/ml gentamicin at the Department of Veterinary Tropical Diseases, University of Pretoria. Asexual spores (conidia) were obtained from 3 day-old culture by flooding the plates with sterile distilled water, pelleted by centrifugation at 3500 x g for 10 min washed in phosphate-buffered saline (0.15 M) and quantified quantified using a haemocytometer [19]. The solution were diluted to 10^7^ spores/100 μL of innoculum.

### Chemotherapeutic trial

Five chicks were randomly selected as a control to rule out prior infection with aspergillus at the beginning of the experiment. These animals were sacrificed, their lungs aseptically removed and infection by *A. fumigatus* was evaluated by placing lung sections onto Sabouraud dextrose agar. The plates were incubated at 37°C and the presence of *A. fumigatus* colonies was checked every day for 1 week [20]. The chicks (n=8) except the neutral group (n=10) were inoculated by transcutaneous injection into the right caudal thoracic air sac with 100 μl spore suspension of a 3-day-old *A. fumigatus* culture containing 10^8^ spores. Birds were observed at least twice a day for the appearance of clinical signs of aspergillosis. Clinical sign of infection (dyspnoea) was evident 3 days post infection. Group 1 served as neutral control and was neither infected nor treated with any substance. Birds in groups 2, 3, and 4 were treated with the extract at 50, 100 and 200 mg/kg, respectively; while groups 5 and 6 were dosed with ketoconazole (60 mg/kg) as the positive control and 0.2% DMSO in water (0.2 ml/100 g) as the untreated control respectively. Ketoconazole was selected as a positive control as it is one of the commonly recommended agents for the treatment of avian aspergillosis [21]. All birds were marked or tagged for identification and were fed non-medicated feed, and clean water was provided freely. All treatments were instituted 3 days post infection and were given intraperitoneally once daily for 3 consecutive days. Birds were subsequently monitored for 12 days from the point of infection.

### Biochemical and haematological analysis

At the end of toxicity and the chemotherapeutic trials, a single blood samples (2.5ml) was collected from each birds from the wing vein or by jugular venipuncture and transferred equally into vacutainer tubes with or without heparin as anticoagulant. Blood samples were centrifuged at 1200 *x g* for 15 minutes in a refrigerated centrifuge (4°C) to separate serum. Alanine aminotransferase (ALT), aspartate aminotransferase (AST) and γ-glutamyltransferase (GGT) were measured using Alfa Wassermann. Total protein, albumin, calcium and phosphorous concentrations were measured using NExCT™ Total Protein reagent, NExCT™ Albumin reagent, ACE™ Calcium-Arsenazo reagent and Alfa Wassermann Inorganic Phosphorus reagent, respectively. Serum globulin levels were deduced by subtracting the albumin levels from the total protein levels [22], and the albumin/globulin (A/G) ratio calculated. The analyses were performed using the ACE TM and NEXT TM Clinical Chemistry Systems (ALFA Wassermann kit, Bayer Health Care, and Johannesburg). Heparinized blood samples were analysed for haemoglobin (Hb), red cell count (RCC), haematocrit (HT), mean corpuscular volume (MCV), mean corpuscular haemoglobin (MCH), mean corpuscular haemoglobin concentration (MCHC) and red cell distribution width (RDW) using ADVIA 2120 haematology analyser (ADVIA, Bayer Health Care, and Johannesburg). White cell count (WCC), absolute neutrophil (total), absolute neutrophil (matured), absolute neutrophil (immatured), absolute lymphocyte, absolute monocyte, absolute eosinophil, absolute basophil analyses were done using manual counter.

### Mycological and pathologic analysis

Birds that died during the toxicity trial were examined by examining the lungs, airsacs, liver, heart, kidneys, spleen and brain observed for gross lesions. The organs were later fixed in 10% formaldehyde. Sections of the organs were embedded in paraffin wax, sectioned at 4 μm and stained with haematoxylin-eosin stain for histopathological examination [20]. Birds that survived were euthanized in a CO_2_ chamber and similarly examined.

The lungs, air sacs and liver of birds from scheduled and unscheduled deaths were specifically evaluated for aspergillosis related lesions. The tissues were aseptically removed and individually plated on SDA using sterile swabs. Plates were incubated for 48 hours at 37°C. When fungal colonies developed, species identification was done by microscopic examination of conidiophores and conidia, in addition to the observation of colony morphology. *A. fumigatus* is characterized by green echinulate conidia, 2 to 3 μm in diameter, produced in chains from greenish phialids, 6 to 8 μm by 2 to 3 μm in size [21]. A portion of lung was also fixed for histopathology as for the toxicity study. The lungs were, however, specifically stained with Grocott methenamine (hexamine) silver for further examination of *Aspergillus fumigatus* hyphae in the tissue section to rule out accidental contamination of samples during collection [22].

### Evaluation of healing effect

At the end of the monitoring period, the organism was recovered from infected chicks and the severity of infection was graded. Microbiological severity of infection was evaluated by adding up the total area covered by individual colony of *Aspergillus fumigatus* on the SDA plate by grade and the total number of colonies present. The grade used: no growth: grade 0; colonies < 2 mm^2^: grade 1; colonies 2–10 mm^2^: grade 2; colonies 10–20 mm^2^: grade 3, colonies > 20 mm^2^: grade 4. Pathological lesion scores were based on the grading system proposed by Delap et al. (1989)[23]. Briefly, the grading is done as follows: grade 0: negative (no lesion), grade 1: localized plaque, grade 2: discrete plaque-moderate, grade 3: discrete plaque that is extensive, grade 4: confluent plaques.

### Statistical analysis

Data obtained from the re-isolation steps of *A. fumigatus* and the sum of area of growth (i.e. by total colony surface area) in infected and treated groups were expressed as mean ± S.E.M. Difference between the groups was analyzed using one way analysis of variance (ANOVA). Results were considered significant if p≤0.05. A post-hoc Dunnett test was used to test for differences to the control for which ANOVA indicated a significant (p≤0.05) F-ratio.

## Results

### Safety evaluation of the extract

**Figure 1:**
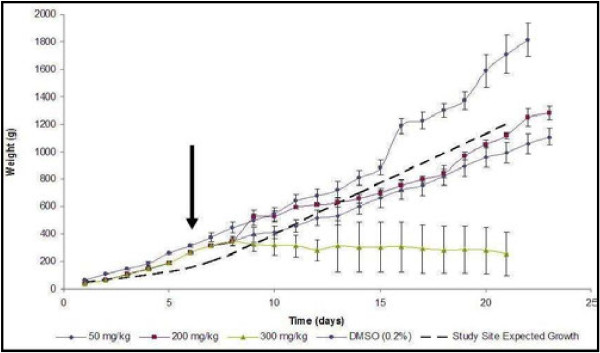
**The effect of crude extract of *L. alata *on weight of broiler chicks.** Arrow indicates time of extract administration. The study site expected growth was derived from historic data for other control animals raised under the same housing and feeding conditions.

Chicks treated with 50 and 200 mg/kg of the extract did not exhibit any clinical sign of toxicity and remained apparently healthy throughout the experimental period. However, at the dose of 300 mg/kg (starting dose), depression, decrease feed intake, diarrhoea, marked weight loss were noticed. One chick died 4 days post treatment (p.t.). In addition, there was marked increased in weight in the DMSO (control) group compared with chicks treated with 50 and 200 mg/kg of the extract. The effect of the extract on weight changes of the birds is presented in Figure 1. The extract caused decreased weight gain when administered at 300 mg/kg. Results pertinent for the serum biochemical profile and haematological analysis conducted at the end of the toxicity trial are presented in Table 1 and Table 2, respectively. Only the globulin concentration of chicks treated with 300 mg/kg of the extract differ significantly (p ≤ 0.05) from other groups. Haematologically, the white cell count (WCC), absolute neutrophil (total and immatured) and absolute lymphocyte of chicks treated with the extract at 200 mg/kg differed significantly (p ≤ 0.05).

**Table 1:** **Biochemical indices of broiler-chicks given varying doses of crude extract of *Loxostylis Alata *in the toxicity study**

**Serum biochemistry**	**Units**	**Dose administered (mg/kg)**			
		**Control (DMSO)**	**50**	**200**	300*
Total serum protein (TSP)	g/l	28.53 ± 1.39	27. 13 ± 2.20	34. 43 ± 2.89	27.90 ± 0.35
Albumin (Alb)	g/l	16. 67 ± 0.58	15.03 ± 0.61	15.50 ± 1.53	15.50 ± 0.55
Globulin (Glob)	g/L	11.87 ± 0.83	12.10 ± 1.59	15.93 ± 3.88	6.70 ± 0.67^a^
Albumin/globulin ratio (A/G)		1.41 ± 0. 05	1.23 ± 0.12	0.96 ± 0.03	2.34 ± 0.01^a^
Calcium (Ca)	mmol/l	2.89 ± 0.03	2.58 ± 0.02	2.62 ± 0.04	2.60 ± 0.13
Serum inorganic phosphate (SIP)	mmol/l	2.45 ± 0.05	2.16 ± 0.07	1.87 ± 0.04	2.00 ± 0.12
Alanine amino transferase (ALT)	u/l	2.00 ± 0.82	1.33 ± 1.33	0.00 ± 0.00	2.00 ± 0.00
Aspartate amino transferase( AST)	u/l	176.67 ± 4.77	160.67 ± 13.61	148.00 ± 9.88	169.5 ± 9.53

N=3, except the 300mg group where one of the chicks died 4-days p.t.

**Table 2:** **Haematological indices of broiler-chicks given varying doses of crude extract of *Loxostylis Alata *in the toxicity study**

**Haematology**	**Units**	**Dose administered (mg/kg)**			
		**Control (DMSO ml/kg)**	**50**	**200**	**300**
Haemoglobin (Hb)	g/dl	143.33 ± 4.7	139.33 ± 0.88	144.67 ± 8.66	135.50 ± 1.50
Red cell count (RCC)	x10^12^/L	2.62 ± 0.12	2.50 ± 0.11	2.83 ± 0.09	2.40 ± 0.02
HT (haematocrit)	l/l	0.36 ± 0.01	0.34 ± 0.01	0.33 ± 0.03	0.30 ± 0.01
Mean cell volume (MCV)	Fl	140.00 ± 2.95	135.67 ± 1.77	126.33 ± 6.90	135.00 ± 1.00
Mean cell haemoglobin (MCH)	Pg	54.77 ± 0.84	54.17 ± 0.54	51.40 ± 3.22	55.10 ± 0.35
Mean cell haemoglobin concentration (MCHC)	g/dl	39.27 ± 0.23	39.23 ± 0.84	40.87 ± 0.63	40.90 ± 0.05
Red cell distribution width (RDW)		13.47 ± 0.923	12.47 ± 0.67	14.77 ± 1.76	13.70 ± 1.15
White cell count (WCC)	x10^9^/L	3.73 ± 0.95	5.33 ± 1.09	19.80 ± 8.06^a^	4.40 ± 0.80
Absolute neutrophil (total)	x10^9^/L	1.31 ± 0.26	1.94 ± 0.47	10.31 ± 4.77^a^	2.30 ± 0.45
Absolute neutrophil (matured)	x10^9^/L	1.31 ± 0.26	1.94 ± 0.47	10.31 ± 4.77^a^	2.30 ± 0.45
Absolute neutrophil (immatured)	x10^9^/L	0.00 ± 0.00	0.00 ± 0.00	0.00 ± 0.00	0.00 ± 0.00
Absolute lymphocyte	x10^9^/L	1.99 ± 0.50	2.56 ± 0.49	6.25 ± 1.66^a^	1.50 ± 0.34
Absolute monocyte	x10^9^/L	0.35 ± 0.15	0.34 ± 0.05	1.83 ± 1.40	0.40 ± 0.16
Absolute eosinophil	x10^9^/L	0.00 ± 0.00	0.27 ± 0.11	0.88 ± 0.03	0.1 ± 0.03
Absolute basophil	x10^9^/L	0.09 ± 0.06	0.07 ± 0.04	0.42 ± 0.02	0.00 ± 0.00

Means with superscript letter differ significantly (p ≤ 0.05) from the control.

N=3, except the 300mg group where one of the chicks died 4-days p.t.

**Figure 2:**
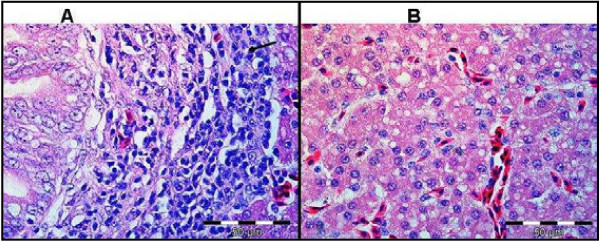
**Liver tissue of birds treated with 300 mg/kg of extract (A) showing scattered bile duct proliferation, focal periductular fibrosis and lymphocytes accumulation (arrow).** Normal liver tissue (B) is shown. H&E.

**Figure 3:**
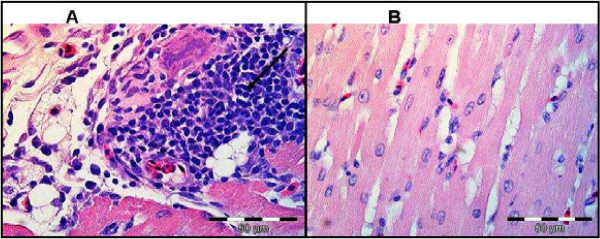
**The heart tissue of birds treated with 300 mg/kg of extract (A) showing lympoplasmacytic pericarditis (arrow).** Normal heart tissue (**B**) is shown. H&E.

**Figure 4:**
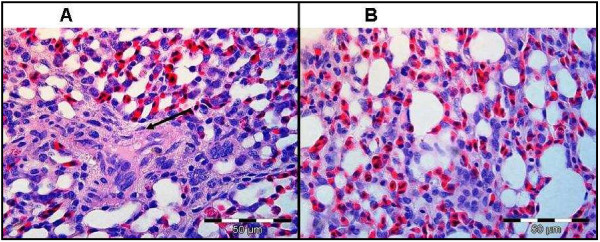
**The lungs tissue of birds treated with 300 mg/kg of extract showing moderate fibrinopurulent bronchitis (arrow). **Normal lung tissue (**B**) is shown. H&E.

Gross post-mortem results showed moderate ascites, hydropericardium, lung oedema and, soft and pliable kidneys in the chick that died from group treated with 300 mg/kg. The remaining two chicks that were sacrificed at the end of the experiment from the same group showed similar pathological signs. Birds treated with 50 and 200 mg/kg of the extract showed no gross pathological signs. Birds treated with 300 mg/kg had histological lesions i.e. scattered bile duct proliferation, focal periductular fibrosis and lymphocytes accumulation in the liver tissues (Figure 2). The heart and lungs had lymphoplasmacytic pericarditis and moderate fibrinopurulent bronchitis (Figures 3 and 4), respectively. No changes were apparent in the organs of birds treated with 50 and 200 mg/kg of the extract.

### Chemotherapeutic trial

#### Clinical signs and survivability

Chicks in the non-treated control groups developed clinical signs 3 days post infection (p.i), with one chick (out of eight) in the group exhibited mild respiratory distress, ruffled feathers, weakness and diarrhoea. The birds in the non-infected and non-treated (negative) group remained apparently normal throughout the experimental period. The effect of the extract on survival of chicks infected with *Aspergillus fumigatus* is presented on Table 3, in what appears to be a dose-dependent protection against death in chicks infected with *Aspergillus fumigatus*. The effect seen was similar to that for the commercial ketoconazole antifungal positive control.

**Table 3:** **The effect of the extract doses of crude extract of *Loxostylis Alata *on survival of chicks infected with *Aspergillus Fumigatus***

**Dose (mg/kg)**	**No. of chicks**	**No. survived**	**No. death**	**% Mortality**
0	8	2	6	75
50	8	3	5	62.5
100	8	3	5	62.5
200	8	6	2	25
Ketoconazole (60 mg/ml)	8	7	1	12.5
Neutral	10	8	0	0

#### Macroscopic lesions

**Figure 5:**
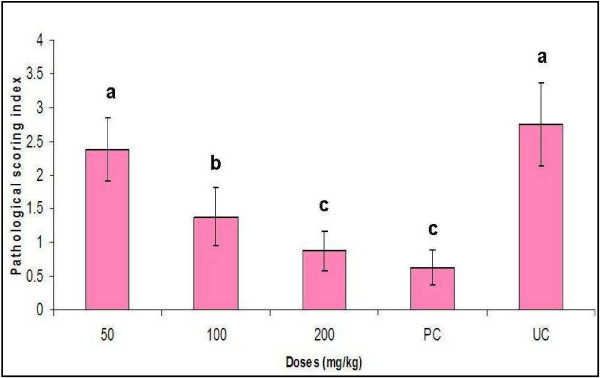
**Effect of different doses of *L. alata *extract on pathological lesions in the lungs, airsac and liver of broiler chicks caused by *A. fumigatus***. Values are mean ± SEM. Means with different letter alphabet differ significantly with control and the other treatment groups (p ≤ 0.05). No pathological lesion was recorded in the uninfected and untreated control chicks.

Gross lesions were detected at necropsy in all the infected-treated birds. Lesions consisted of small (1 to 3 mm) white nodules on the surface of the lungs and liver. Cloudiness of the thoracic air sacs was also noticed. However, the severity of the lesions was lower in chicks treated with the extract and also for the positive control group (ketoconazole treated chicks). The extract, therefore reduced the severity of the lesions in what appears to be dose-dependent (Figure 5).

#### Mycological cultures

**Figure 6:**
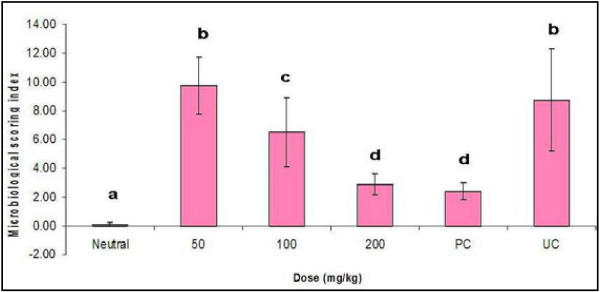
**Re-isolation of *A. fumigatus *from the lung, airsac and liver of infected broiler chicks treated with different doses of *L. alata.*** Values are mean ± SEM. Means with different letter alphabet differ significantly (p ≤ 0.05) for the control and the other treatment groups.

No *A. fumigatus* was isolated from the 5 chicks sacrificed at the start of chemotherapeutic trial. In the infected and treated groups varying amounts of *A. fumigatus* colonies were isolated from the airsac, lungs and liver tissue. In addition, *A. fumigatu*s was also isolated from the lung sample of one of the chicks in the neutral control group (Figure 6) indicating a subsequent contamination.

#### Special histological evaluation of lungs

**Figure 7:**
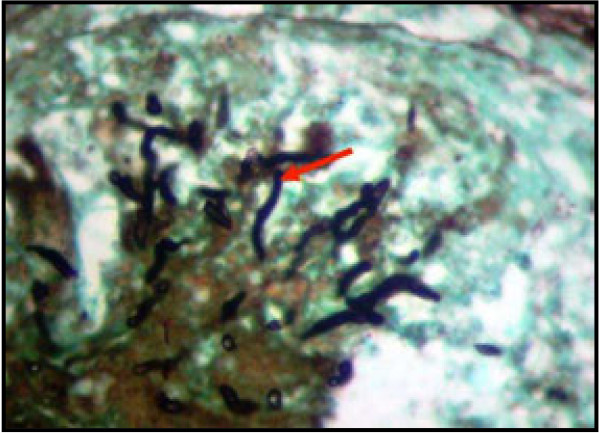
***Aspergillus fumigatus *hyphae (arrow) radiating in a pulmonary lobule of one of the infected chicks stained with Grocott methenamine (hexamine) silver (X1000).**

Sections of lungs from infected chicks showed the presence of fungal hyphae when special staining method was used. However, no fungal hyphae were noted in the lung samples of chicks in the neutral control group. Figure 7 showed the presence of small hyphae of *A. fumigatus* radiating in a pulmonary lobule.

#### Biochemical and haematological profile

Results for the serum and blood analysis of chicks infected with *A. fumigatus* are presented in Tables 4 and 5, respectively. There was a significant (p ≤ 0.05) increase in the concentration of aspartate amino transferase and globulin in chicks that received DMSO. Likewise, serum gamma glutamyl transferase (GGT) were markedly (p ≤ 0.05) increased in chicks treated with ketoconazole and DMSO. Haematologically, the white cell count (WCC), absolute neutrophil (total and matured) and absolute monocytes (results not shown) in chicks treated with the extract at 200 mg/kg and ketoconazole do not differ significantly (p ≤ 0.05), however, they increase markedly (p ≤ 0.05) in untreated control groups.

**Table 4:** **Biochemical indices of broiler-chicks infected with *A. Fumigatus *and treated varying doses of crude extract of *Loxostylis Alata***

**Serum biochemistry**	**Units**	**Dose administered (mg/kg)**					
		**Neutral**	**50**	**100**	**200**	**PC (60)**	**UC**
Total serum protein (TSP)	g/l	25.52 ± 0.48	26.67 ± 0.90	28.98 ± 0.74	30.07 ± 2.26	30.90 ± 2.03	36.05 ± 9.15
Albumin (Alb)	g/l	14.33 ± 0.26	14.43 ± 0.12	15.98 ± 0.62	15.33 ± 0.39	15.90 ± 0.17	15.60 ± 0.00
Globulin (Glob)	g/L	11.19 ± 0.30	12.23 ± 0.87	13.00 ± 0.35	14.74 ± 2.34	15.00 ± 1.93	24.50 ± 9.20^a^
Albumin/globulin ratio (A/G)		1.29 ± 0.03	1.19 ± 0.09	1.23 ± 0.06	1.13 ± 0.10	1.15 ± 0.12	0.61 ± 0.43^a^
Calcium (Ca)	mmol/l	3.29 ± 0.10	3.42 ± 0.10	3.53 ± 0.14	3.37 ± 0.16	3.40 ± 0.10	2.92 ± 0.07
Serum inorganic phosphate (SIP)	mmol/l	1.43 ± 0.08	1.59 ± 0.10	1.60 ± 0.10	1.66 ± 0.10	1.65 ± 0.11	2.38 ± 0.96
Alanine amino transferase (ALT)	u/l	3.00 ± 0.61	3.33 ± 1.67	2.80 ± 0.97	4.86 ± 2.58	1.71 ± 0.57	1.50 ± 1.50
Aspartate amino transferase( AST)	u/l	149.20 ± 4.08	153.00 ± 8.54	138.60 ± 7.26	155.00 ± 6.24	129.43 ± 9.86	171.50 ± 22.20^a^
γ-glutamyltransferase (GGT)	u/l	19.60 ± 0.50	22.67 ± 0.88	23.00 ± 2.45	24.14 ± 4.12	26.57 ± 3.09	36.50 ± 17.50^a^

Neutral group are chicks that were not infected with *A. fumigatus*, and were also not treated. Positive control (PC) (ketoconazole); Untreated control (UC).

Means with superscript letter differ significantly (p ≤ 0.05) from the control.

**Table 5:** **Haematological indices of broiler-chicks infected with *A. Fumigatus *and treated varying doses of crude extract of *Loxostylis Alata***

**Serum haematology**	**Units**	**Dose administered (mg/kg)**					
		**Neutral**	**50**	**100**	**200**	**PC (60)**	**UC**
Haemoglobin (Hb)	g/dl	131.60 ± 1.93	134.33 ± 4.10	140.00 ± 2.92	133.14 ± 2.38	141.71 ± 5.97	138.00 ± 1.00
Red cell count (RCC)	x10^12^/L	2.43 ± 0.40	2.48 ± 0.11	2.60 ± 0.06	2.56 ± 09	2.70 ± 0.18	2.77 ± 0.10
HT (haematocrit)	l/l	0.34 ± 0.10	0.34 ± 01	0.35 ± 0.01	0.34 ± 01	0.36 ± 02	0.35 ± 0.01
Mean cell volume (MCV)	Fl	138.10 ± 1.06	139.00 ± 1.15	136.40 ± 3.75	134.43 ± 2.10	134.71 ± 3.31	125.50 ± 6.50
Mean cell haemoglobin (MCH)	Pg	54.14 ± 0.45	54.33 ± 0.94	53.80 ±0.86	52.29 ± 1.06	52.93 ± 1.40	50.00 ± 2.30
Mean cell haemoglobin concentration (MCHC)	g/dl	39.17 ± 0.28	39.10 ± 0.53	39.52 ± 0.52	38.90 ± 0.35	39.33 ± 0.29	39.95 ± 0.25
(Red cell distribution width) RDW		12.83 ± 0.13	13.53 ± 0.38	13.40 ± 0.57	14.07 ± 0.41	13.93 ± 1.04	14.30 ± 1.40
White cell count (WCC)	x10^9^/L	15.54 ± 2.13	18.80 ± 1.50	16.60 ± 3. 18	28.29 ± 6.60^a^	29.43 ± 10.32^a^	49.00 ± 13.21^b^
Absolute neutrophil (total)	x10^9^/L	4.71 ± 0.96	8.82 ± 1.04	6.71 ± 2.58	13.04 ± 4.52^a^	17.79 ± 7.65^a^	26.28 ± 16.36^b^
Absolute neutrophil (mature)	x10^9^/L	4.71 ± 0. 96	8.82 ± 1.04	6.71 ± 2.58	13.04 ± 4.52^a^	17.79 ± 7.65^a^	26.28 ± 16.36^b^
Absolute neutrophil (immature)	x10^9^/L	0.00 ± 00	0.00 ± 0.00	0.00 ± 0.00	0.00 ± 0.00	0.00 ± 0.00	0.00 ± 0.00

Neutral group are chicks that were not infected with *A. fumigatus*, and were also not treated. Positive control (TC); Untreated control (UC).

Means with differing superscript letters in the same row are significantly different (p ≤ 0.05).

## Discussion

Generally, aspergillosis in chickens is an acute disease causing high mortality in the first few days of life. The disease in some cases can be chronic, with lesions in the lungs, air sacs, and joints [3]. Evidence of clinical signs associated with *A. fumigatus* infection was quite evident in this study, which is in agreement with literature. Our data indicated that the crude extract of *L. alata* has certain level of activity against aspergillosis in broiler chicks and could serve as model for the development of safer and effective anti-Aspergillus agents. Plant drugs have been assumed to be safe. However, in recent times, there is evidence that many plants used as food or as medicine can be potentially toxic [24,25]. All drugs can produce harmful as well as beneficial effects, hence the need for toxicity testing of any substance intended for development into drug for human or animal use [26]. Investigation of the acute toxicity is the first step in the toxicological investigations of an unknown substance [27]. The index of the acute toxicity is the lethal dose 50 (LD_50_). However, the LD_50_ is not regarded as a biological constant, since differing results are obtained on repetition [27]. The OECD guidelines for testing chemicals (2000) for toxicity were adapted in testing the safety of the extract of *Loxostylis alata*[28]. The method is not intended to allow the calculation of a precise LD_50_, but does allow for the determination of defined exposure ranges where lethality is expected since death or appearance of toxic signs of a proportion of the animals is still the major endpoint of this test. The extract was toxic at the dose of 300 mg/kg based on its effects on different parameters measured and clinical signs noticed on the experimental chicks. Dose is an important factor in drug poisoning and most drugs cause clinical signs of poisoning when administered at relatively high dose [26].

At lower doses (200 mg/kg and below) the extract had no apparent toxic effects. Although, the chicks treated with 50 and 200 mg/kg had lower weight gain than those in the control group, their weights were within the normal growth rate for broiler chickens expected for the particular research site and reported in literature [29,30]. The reason for the DMSO treated healthy birds, on average, out-performing the historical growth reported for the facility is not known at this stage.

Clinical signs of aspergillosis observed in our study included ruffled feathers, gasping, dyspnoea, dullness, green watery diarrhoea and anorexia. These signs are in agreement and confirmed the clinical signs observed in previous studies [3]. Although ascites and blindness were observed by Julian and Goryo (1990) and Akan et al. (2002), respectively, we did not observe these clinical features in our study [31,32]. Chicks in this study started dying 3 days p.i. with mortality reaching as up to 75% in the non-treated group. Furthermore, *A. fumigatus* was re-isolated from the lungs and airsacs of infected chicks from 3 days p.i up to 12 days p.i. Lesions were seen mainly in the lungs, liver and the airsac. From a clinical efficacy point, the presence of organisms in the lungs after treatment, with either the plant extract or the ketoconazole, may be seen as therapeutic failure. However the failure to clear the organism is most likely linked to the short duration of treatment as opposed to product failure. As such for future studies, it is recommended that treatment extend for longer periods perhaps daily for 2 weeks.

Surprisingly, *A. fumigatus* was isolated from the lung sample of one chick in the neutral group, which was not infected and also not treated. Since systemic infection with *Aspergillus fumigatus* results naturally through the inhalation of spores, [32] it is possible that the chicks in the neutral group were infected naturally from fungal spores inhaled in the air. However, we used a specific histological staining technique to confirm the presence of an infection, and are therefore certain that the positive result was due to contamination during post mortem examination.

Increase AST and GGT activity as well as A/G ratio are indicative of a liver injury. This was supported by the isolation of the agent from liver. In addition, there was presence of lesions on the liver. Compared with the infected untreated group with a mortality rate of 75%, in the entire infected treated group, the mortality rate reduced and death was stopped on 11th day post-infection. Treatment with extract of *Loxostylis alata* at doses used confer a dose related success in combating infection due to *A. fumigatus* in broiler chicks and that compared favourably with the reference compound (ketoconazole). Compounds administered i.p. are absorbed primarily through the portal circulation and, therefore**,** must pass through the liver before reaching other organs. This factor can limit the amount of drug reaching its site of action [33]. Indeed, there are numerous examples in the literature of drugs that are less effective after i.p. administration than when given by other parenteral routes. Such examples include reserpine [34], phenelzine and phenipramine [35]. It will therefore be reasonable to assume that when the extract is administered via another route that bypasses the portal circulation could result in enhanced action of the extract.

## Conclusions

In conclusion, broiler chicks are susceptible to the infection with *A. fumigatus* and the extract of *L. alata* appears to be beneficial in treating and curing it. Additional work is needed on extracts of this species to further establish its detailed safety and efficacy against *A. fumigatus* in broiler chicks and other avian species.

## Competing interests

The authors declare that they have no competing interests.

## Authors’ contributions

MMS, VN: Conceived, designed and undertook experiment; ND: Necropsy and histopathological evaluations; VN: Statistical analysis; MMS, JNE, VN: Interpreted the study data, wrote and edited the manuscript. All authors have read and approved the final version of the manuscript.
